# Loss of sphingosine 1-phosphate (S1P) in septic shock is predominantly caused by decreased levels of high-density lipoproteins (HDL)

**DOI:** 10.1186/s40560-019-0376-2

**Published:** 2019-04-17

**Authors:** Martin Sebastian Winkler, Konstantin B. Märtz, Axel Nierhaus, Günter Daum, Edzard Schwedhelm, Stefan Kluge, Markus H. Gräler

**Affiliations:** 1 Department of Anesthesiology, University Medical Center Hamburg-Eppendorf, Martinistr. 52, Hamburg, 20246 Germany; 2 Department of Anesthesiology and Intensive Care Medicine, Center for Sepsis Control and Care (CSCC), and the Center for Molecular Biomedicine (CMB), Jena University Hospital, Hans-Knöll-Str. 2, 07745 Jena, Germany; 3 Department of Intensive Care Medicine, University Medical Center Hamburg-Eppendorf, Martinistr. 52, Hamburg, 20246 Germany; 4 Clinic and Polyclinic for Vascular Medicine, University Heart Center, Martinistrasse 52, 20246 Hamburg, Germany; 5 Institute of Clinical Pharmacology and Toxicology, University Medical Center Hamburg-Eppendorf, Martinistr. 52, Hamburg, 20246 Germany; 6 Department of Anesthesiology and Intensive Care Medicine, Universitätsmedizin Göttingen, Robert-Koch-Str. 40, 37075 Göttingen, Germany

**Keywords:** Volume resuscitation, Serum albumin, Sepsis, Sequential Organ Failure Assessment, Endothelial cell barrier

## Abstract

**Background:**

Sphingosine 1-phosphate (S1P) is a signaling lipid essential in regulating processes involved in sepsis pathophysiology, including endothelial permeability and vascular tone. Serum S1P is progressively reduced in sepsis patients with increasing severity. S1P function depends on binding to its carriers: serum albumin (SA) and high-density lipoproteins (HDL). The aim of this single-center prospective observational study was to determine the contribution of SA- and HDL-associated S1P (SA-S1P and HDL-S1P) to sepsis-induced S1P depletion in plasma with regard to identify future strategies to supplement vasoprotective S1P.

**Methods:**

Sequential precipitation of lipoproteins was performed with plasma samples obtained from 100 ICU patients: surgical trauma (*n* = 20), sepsis (*n* = 63), and septic shock (*n* = 17) together with healthy controls (*n* = 7). Resultant fractions with HDL and SA were analyzed by liquid chromatography coupled to triple-quadrupole mass spectrometry (LC-MS/MS) for their S1P content.

**Results:**

Plasma S1P levels significantly decreased with sepsis severity and showed a strong negative correlation with increased organ failure, quantified by the Sequential Organ Failure Assessment (SOFA) score (rho − 0.59, *P* < 0.001). In controls, total plasma S1P levels were 208 μg/L (187–216 μg/L). In trauma patients, we observed an early loss of SA-S1P (− 70%) with a concurrent increase of HDL-S1P (+ 20%), resulting in unaltered total plasma S1P with 210 μg/L (143–257 μg/L). The decrease of plasma S1P levels with increasing SOFA score in sepsis patients with 180.2 μg/L (123.3–253.0 μg/L) and in septic shock patients with 99.5 μg/L (80.2–127.2 μg/L) was mainly dependent on equivalent reductions of HDL and not SA as carrier protein. Thus, HDL-S1P contributed most to total plasma S1P in patients and progressively dropped with increasing SOFA score.

**Conclusions:**

Reduced plasma S1P was associated with sepsis-induced organ failure. A constant plasma S1P level during the acute phase after surgery was maintained with increased HDL-S1P and decreased SA-S1P, suggesting the redistribution of plasma S1P from SA to HDL. The decrease of plasma S1P levels in patients with increasing sepsis severity was mainly caused by decreasing HDL and HDL-S1P. Therefore, strategies to reconstitute HDL-S1P rather than SA-S1P should be considered for sepsis patients.

## Background

Sepsis is a life-threatening organ dysfunction caused by a dysregulated host response to infection [1]. Despite many efforts for the development of specific drugs, early antibiotic treatment still remains the only causative treatment option in combination with general life support by vasoactive drugs and volume resuscitation [2]. Crystalloids are recommended by the current Surviving Sepsis Campaign Guidelines as first-line fluids for septic shock resuscitation [3]. The use of human albumin (HA) is still controversial [4]. One argument supporting the reconstitution of HA is the observed better and long-lasting intravascular effect and volume expansion compared to crystalloid fluids mainly via re-establishing the oncotic pressure. Another often discussed argument is that circulatory serum albumin (SA) is an intrinsic and essential carrier for a variety of vasoprotective molecules such as sphingosine 1-phosphate (S1P). S1P regulates many pathophysiological processes responsible for sepsis severity including endothelial barrier protection, lymphocyte trafficking, uncontrolled cytokine secretion, and vascular tone [5].

Currently, four observational studies show that S1P levels are severely compromised in serum and plasma of septic patients [6–9], and experimental data in septic animals have shown that intravenous supplementation of S1P is able to dampen sepsis symptoms such as lung edema, capillary leakage, and vasoplegia [10–13]. The main S1P function of endothelial leakage protection has been lately discussed as most relevant in sepsis-induced organ dysfunction [14]. Indeed in humans, low S1P levels are not only indicative of a septic shock with similar potency as the Sequential Organ Failure Assessment (SOFA) [9] but may also serve as a new therapeutic option for sepsis treatment due to the beneficial activities of S1P on the integrity of the endothelial layer and the immune response and as a pro-survival factor [15]. However, how to reconstitute S1P is still controversial.

In circulation, S1P is constitutively produced and released by endothelial cells (EC) and red blood cells (RBC) [16, 17]. Activation-induced S1P release was also reported for other cells including platelets and macrophages [18, 19]. S1P then binds to apolipoprotein M (ApoM) in HDL or is associated with SA as the two major S1P carriers in plasma [20, 21]. HDL has been shown to be the preferred carrier for S1P over SA in plasma [22]. Interestingly, HDL-S1P contributes to the anti-atherosclerotic and EC barrier-stabilizing effects of HDL [23]. A specific functional role of SA-S1P has not yet been described.

This study investigated the contribution of the two major carriers for S1P in plasma, HDL, and SA, to the observed loss of S1P in plasma of patients with septic shock in comparison with healthy volunteers, surgical trauma patients, and sepsis patients.

## Materials and methods

### Study population

The presented data are based on a single-center prospective-observational trial which has been performed from March to December 2014. One hundred patients who were admitted to the intensive care units (ICU) of the University Medical Center Hamburg-Eppendorf (Hamburg, Germany) with sepsis or after surgery were enrolled after informed consent had been obtained from patients or their legal representatives. The study cohort was previously described [9, 24], and this is a follow-up analysis including all 100 patients recruited for the original study. In this study, plasma samples instead of serum samples were analyzed for S1P concentrations present in total plasma and protein fractions enriched for the two major S1P carriers HDL and SA. The parameters were pre-specified in the study protocol approved by the local Research Ethics Committee (Hamburg Chamber of Physicians: reference PV4550). Blood samples from controls and patients were all processed the same way. EDTA-plasma was centrifuged and immediately frozen and stored at − 80 °C until S1P and lipoprotein measurement. The study included patients when diagnosed with an infection or with a clinical syndrome pathognomonic for an infection according to the former sepsis criteria [9]. Since 2016, the Sepsis-3 guidelines were published, and all patients were re-categorized according to the latest Sepsis-3 definition [24]. A SOFA score for each patient was generated, and three groups were defined: Patients admitted to the ICU post-surgery were categorized as “surgical trauma,” patients admitted to the ICU with suspected or diagnosed infections were categorized as “sepsis,” and patients with hypotension requiring vasopressor therapy to maintain mean BP 65 mmHg or greater plus serum lactate concentration greater than 2 mmol/L in spite of adequate fluid resuscitation were categorized as “septic shock” [24]. Patients were included on day 1 after being diagnosed with sepsis, or after surgery. Blood was drawn upon admission to the ICU. Exclusion criteria were age < 18 years, pregnancy, or a moribund status of the disease. Patients with HA used for fluid resuscitation were also excluded.

### Clinical evaluations and assays

SOFA scores were calculated on admission according to guidelines [24]. Within the first 24 h after inclusion, plasma and serum samples were taken to measure S1P and S1P bound to its specific carrier protein HDL (HDL-S1P) and SA (SA-S1P). The Institute of Clinical Chemistry and Laboratory Medicine at the University Medical Center Hamburg-Eppendorf (Hamburg, Germany) measured the concentration of HDL and SA together with all other markers: hemoglobin, hematocrit, platelets, creatinine, leucocytes, lactate, interleukin-6 (IL-6), procalcitonin (PCT), and C-reactive protein (CRP).

### Lipoprotein precipitation

Lipoproteins were sequentially precipitated via an increasing Na_3_P(W_3_O_10_)_4_ concentration as previously described with minor modifications [20, 25]. For chylomicron and very low-density lipoprotein (VLDL) precipitation, 25 μL of 1% Na_3_P(W_3_O_10_)_4_ and 25 μL of a 2 M MgCl_2_ solution were added to 500 μL plasma. After brief mixing and 15 min incubation at room temperature, the samples were centrifuged at room temperature for 10 min at 6000 rcf, and supernatants were transferred to new tubes for further precipitation. For low-density lipoprotein (LDL) precipitation, 25 μL of 4% Na_3_P(W_3_O_10_)_4_ solution was added to the first supernatant. After 15 min incubation at room temperature (RT), the samples were centrifuged at room temperature for 10 min at 6000 rcf, and the supernatants were again transferred to new tubes. For HDL precipitation, 25 μL of 40% Na_3_P(W_3_O_10_)_4_ and 25 μL of a 2 M MgCl_2_ solution were added to the second supernatant. After 2 h incubation at room temperature, the samples were centrifuged at room temperature for 30 min at 20,000 rcf. The supernatant was the lipoprotein-free SA-containing fraction. If not used immediately, the fractions were stored at − 20 °C.

### Extraction and quantification of S1P

S1P measurements were performed according to an established protocol using liquid chromatography coupled to triple-quadrupole mass spectrometry (LC/MS/MS) [26]. After addition of C17-base S1P as internal standard (100 pmol/sample, Avanti Polar Lipids), samples were transferred to glass centrifuge tubes and adjusted to a final volume of 1 mL with H_2_O. After addition of 0.3 mL 6 N HCl, 1 mL methanol, and 2 mL chloroform, samples were vigorously vortexed for 10 min. Aqueous and chloroform phases were separated by centrifugation for 3 min at 1900 rcf, and the lower chloroform phase was transferred into a new glass centrifuge tube. After a second round of lipid extraction with additional 2 mL chloroform per milliliter aqueous sample, the two chloroform phases were combined and vacuum-dried at 60 °C for 50 min using a vacuum concentrator. The extracted lipids were dissolved in 100 μL methanol/chloroform (4:1, *v*/*v*) and stored at − 20 °C. Detection was performed with the QTrap triple-quadrupole mass spectrometer (Sciex) interfaced with the 1100 series chromatograph (Agilent), the Hitachi Elite LaChrom column oven (VWR), and the Spectra System AS3500 autosampler (Thermo Separation Products). Positive electrospray ionization (ESI) LC/MS/MS analysis was used for detection of S1P and C17-S1P. Multiple reaction monitoring (MRM) transitions were as follows: S1P m/z 380/264 and C17-S1P m/z 366/250. Liquid chromatographic resolution of all analytes was achieved using a 2 × 60 mm MultoHigh C18 reversed phase column with a 3-μm particle size (CS-Chromatographie Service). The column was equilibrated with 10% methanol and 90% of 1% formic acid in H_2_O for 5 min, followed by sample injection and 15 min elution with 100% methanol with a flow rate of 300 μL/min. Standard curves were generated by adding increasing concentrations of S1P to 100 pmol of the internal standard C17-S1P. Linearity of the standard curves and correlation coefficients were obtained by linear regression analyses. Data analyses were performed using Analyst 1.6 (Sciex).

### Statistical analysis

The primary variables were plasma S1P, HDL-S1P, and SA-S1P in micrograms per liter. Differences between two groups were tested for significance using the non-parametric Mann-Whitney *U* test or Kruskal-Wallis analysis of variance (ANOVA) for more than two groups. Data are presented as medians with interquartile range (IQR) if not otherwise indicated. Correlations were calculated using Spearman’s rank correlation. The cohort was divided into SOFA tertiles: lower < 3, median 4–7, and upper ≥ 8 SOFA tertiles to demonstrate difference in patients with rising severity. Odds ratios with 95% confidence intervals (CIs) were computed by using a multivariate logistic regression model with septic shock or a SOFA score ≥ 8 as dependent variable. For all tests, a *P* value of less than 0.05 was considered significant. Statistical analyses were performed by using Graph Pad Prism 7.0a, April 2016 (La Jolla, CA, USA) and SPSS (version 21; IBM Corporation, Armonk, NY, USA) with guidance from members of the Department of Medical Biometry and Epidemiology at the University Hospital Hamburg-Eppendorf.

## Results

Plasma S1P levels were measured in a cohort of 100 ICU patients and 7 healthy volunteers. The patients’ cohort has been described and was included in the study following the previous sepsis consensus and the systemic inflammatory response syndrome (SIRS) criteria. The cohort has been rearranged to meet the latest 2016 Sepsis-3 criteria [9, 24]. The surgical trauma group comprised 20 patients admitted to ICU for postoperative monitoring after elective major surgery; these patients had undergone abdominal or thoracic surgery (*n* = 14) or other types of surgery (*n* = 6). The sepsis group comprised 63 patients, and the septic shock group comprised 17 patients (Table 1) [9, 24]. Consistent with the clinical status, the SOFA scores were highest in patients with septic shock. Septic shock patients stayed longer on ICU, were more often mechanically ventilated, or needed dialysis more often (Table 1).

**Table 1:** Characteristics of study cohort

Clinical parameter	Control	Surgical trauma	Sepsis	Septic shock	*P* value^*^
*n*	7	20	63	17	N/A
Age, years	31 (24–52)	61 (51–68)	60 (49–70)	60 (54–72)	ns
Male, *n* (%)	3 (42)	11 (55)	37 (62)	10 (58)	ns
SOFA score	N/A	4 (2–7)	5 (3–7)	11 (8–13)	< 0.0001
Leucocytes, ×10^9^/L	6.3 (5.8–7.9)	13.0 (12.0–15.1)	14.0 (9.9–18.5)	14.6 (9.4–24.9)	ns
Interleukin-6, ng/L	N/D	129.4 (61.7–263.3)	123.4 (46.2–304.5)	464.9 (132.7–1568)	< 0.05^#^
Procalcitonin, μg/L	N/D	0.81 (0.28–3.06)	0.73 (0.25–1.76)	4.19 (1.53–9.27)	< 0.01^#^
C-reactive protein, mg/L	5.0 (5.0–5.5)	97.5 (68.2–190.8)	145 (72.0–239.0)	128 (100.5–222.0)	ns
Lactat, mmol/L	N/D	1.2 (0.92–1.7)	1.3 (1.0–1.6)	2.8 (2.2–5.4)	< 0.0001
ICU length of stay, days	N/A	2 (1–5)	7 (3–10)	13 (8–31)	< 0.0001
Invasive ventilation, *n* (%)	N/A	1 (5)	19 (30)	8 (47)	< 0.01^#^
Dialysis, *n* (%)	N/A	0 (0)	4 (6)	6 (35)	< 0.001^#^
Died, *n* (%)	N/A	0 (0)	10 (16)	5 (30)	< 0.05^#^

*SOFA* Sequential Organ Failure Assessment score, *ICU* intensive care unit, *N/A* not applicable, *N/D* not determined, *ns* not significant. Data are presented as median (IQR). ^*^P value for trend between patient groups using a non-parametric ANOVA Kruskal-Wallis test if not otherwise indicated. ^#^Chi-square for trend analysis

### Plasma S1P levels significantly decreased in patients with septic shock

**Fig. 1:**
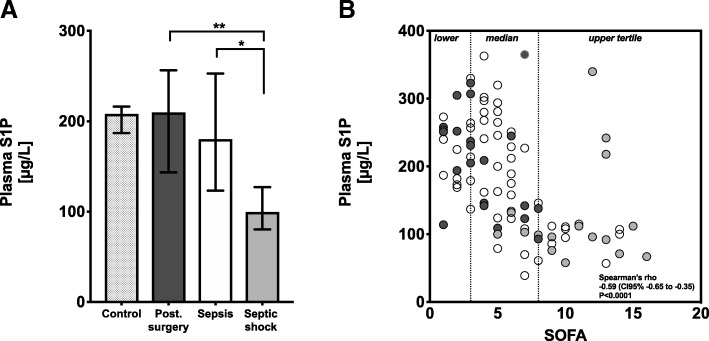
Plasma sphingosine 1-phosphate (S1P) concentrations in patient groups (**a**) and correlation with SOFA score (**b**). **a** Plasma S1P levels are presented as median with interquartile range, and patient groups were compared using a non-parametric ANOVA Kruskal-Wallis test between groups. Plasma S1P levels are lowest in patients with septic shock. *P < 0.05; **P < 0.01. **b** Plasma S1P levels are plotted together with the SOFA score. Surgical trauma patients: dark gray; sepsis patients: white; and septic shock patients: light gray dots. Vertical dotted lines indicate SOFA tertiles: lower SOFA ≤ 3, median SOFA 4–7, and upper SOFA ≥ 8 tertile. The statistic represents Spearman’s rank correlation for plasma S1P levels and the SOFA score with rho and 95% confidence interval

The median plasma S1P concentration in patients with surgical trauma was 210 μg/L and not different from healthy controls (Fig. 1a, Table 2). The trend towards decreased plasma S1P levels from trauma to septic shock was significant (*P* < 0.01; Table 2). Group comparison showed that plasma S1P levels in sepsis patients were not significantly altered with 180 μg/L compared to controls and surgical trauma patients. Only septic shock patients had significantly reduced total plasma S1P levels with 99 μg/L (Fig. 1a, Table 2). To demonstrate a potential association with the severity of sepsis, which is best described by the sepsis defining SOFA score, plasma S1P levels were correlated with the SOFA score. As a result, plasma S1P levels inversely correlated with the SOFA score with a Spearman’s rho (rho) of − 0.59 (Fig. 1b; *P* < 0.0001).

**Table 2:** Carrier proteins and fractions of sphingosine 1-phosphate in patient groups

Variable	Control	Surgical trauma	Sepsis	Septic shock	*P* value^*^
Carrier proteins of sphingosine 1-phosphate					
HDL, mg/dL	60.0 (53.0–64.0)	32.0 (22.0–45.2)	31.0 (18.2–40.7)	16.0 (12.0–19.0)	< 0.01
SA, g/L	41.0 (40.0–45.0)	21.0 (18.0–23.0)	21.0 (15.0–26.2)	17.0 (13.0–21.0)	ns
Fractions of sphingosine 1-phosphate					
Total plasma S1P, μg/L	208.1 (186.7–216.3)	209.7 (143.3–256.6)	180.2 (123.3–253.0)	99.5 (80.2–127.2)	< 0.01
HDL-S1P, μg/L *percent of total, %*	97.9 (83.5–111.6) *45.5 (44.1–47.4)*	116.5 (82.3–155.4) *59.1 (49.6–64.4)*	100.9 (71.2–137.7) *54.8 (48.4–64.9)*	54.2 (42.8–86.7) *53.8 (44.0–62.7)*	< 0.001
SA-S1P, μg/L *percent of total, %*	72.2 (61.3–75.9) *35.1 (32.4–35.6)*	19.6 (7.6–31.4) 11.1 (4.5–13.6)	15.6 (8.1–33.6) *8.8 (5.2–15.7)*	6.5 (3.5–13.6) *5.7 (4.6–12.1)*	< 0.001

*HDL* high-density lipoprotein, *SA* serum albumin, *S1P* sphingosine 1-phosphate, *ns* not significant. Data are presented as median (IQR). ^*^P value for trend between all groups using a non-parametric ANOVA Kruskal-Wallis test

### S1P shifted from SA to HDL in surgical trauma patients with unchanged total plasma S1P

**Fig. 2:**
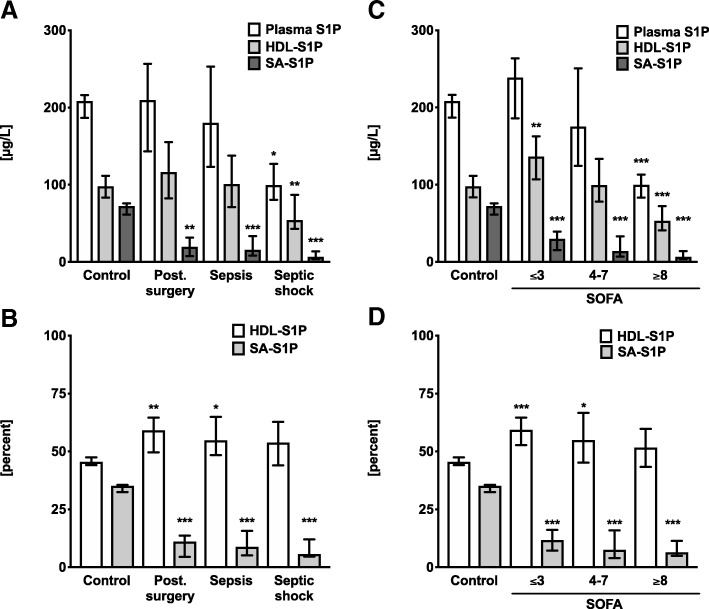
Total plasma sphingosine 1-phosphate (S1P) plotted together with S1P associated with serum albumin (SA-S1P) and S1P bound to high-density lipoprotein (HDL-S1P). **a** S1P in patient groups in micrograms per liter and **b** in fractions of total plasma S1P (percent). **c**, **d** To demonstrate levels and fractions related to severity of sepsis, the cohort was adjusted according to individual SOFA scores. Groups are compared regarding to SOFA tertiles, which are generated from 100 ICU patients: Lower SOFA ≤ 3, median SOFA 4–7, and upper SOFA ≥ 8 tertile. Groups are compared using S1P concentrations in micrograms per liter (**c**) and in fractions of total plasma S1P (**d**). Data are presented as median and 95% confidence interval. Groups are compared using a non-parametric Mann-Whitney *U* test against control: *P < 0.05; **P < 0.01; ***P < 0.001

In plasma, S1P is mainly bound to two carrier proteins: HDL and SA. Therefore, we were interested if the association of S1P with these two carriers was altered in patients. Surprisingly, SA-S1P levels were already significantly reduced in surgical trauma patients compared to healthy controls by more than 70% (Fig. 2a, Table 2). In contrast, the relative contribution of HDL-S1P to total plasma S1P was significantly increased by about 20% (Fig. 2b, Table 2). This shifted contribution of S1P from SA to HDL was even more significant when patients were re-grouped according to the SOFA score tertiles (Fig. 2c, d). Importantly, despite the major decrease of SA-S1P in surgical trauma patients, total plasma S1P levels did not change between controls and surgical trauma patients due to the increased association of S1P with HDL (Fig. 2a–d).

### Decreased HDL-S1P levels provoked low plasma S1P concentrations with increasing organ failure

The contribution to total plasma S1P levels shifted from originally 35 to 11% of SA-S1P, but 46 to 59% HDL-S1P when comparing controls with surgical trauma patients characterized by low SOFA score. Sepsis patients with a medium SOFA score of 4–7 showed a similar loss of both, total plasma S1P and HDL-S1P by 15%, which was not significant. However, in septic shock patients with highest SOFA scores above 8, total plasma S1P and HDL-S1P levels were even further reduced by 45%, which was statistically significant (Fig. 2a, c and Table 2). Notably, although SA-S1P levels were also further reduced to 9% of total plasma S1P levels in sepsis patients and 6% in septic shock patients, the overall contribution to total plasma S1P was already extremely low. The impact of this relative decrease of SA-S1P was negligible compared to the differences in HDL-S1P, which maintained a contribution of more than 50% to total plasma S1P experiments across all patient groups (Fig. 2b, d).

### High SOFA scores were associated with decreased HDL carrier, but not SA carrier

**Fig. 3:**
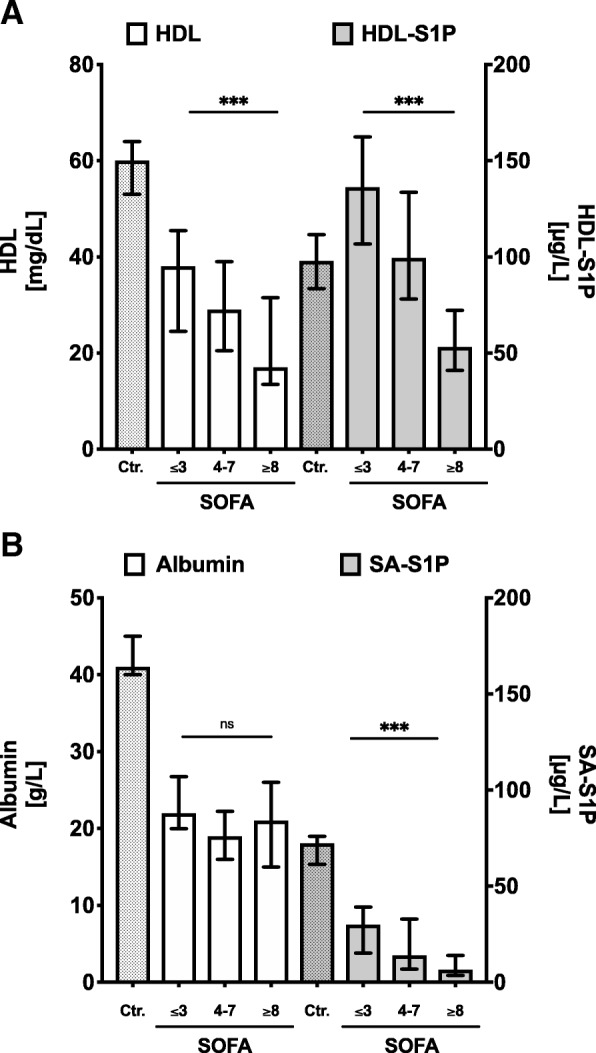
Comparison of carrier proteins of sphingosine 1-phosphate (S1P, left *y*-axis) and carrier-specific S1P fraction (right *y*-axis) in sepsis patients with rising severity. **a** High-density lipoprotein (HDL) and HDL-S1P and **b** serum albumin (SA) and SA-S1P are shown. Data are presented as median and 95% confidence interval. Patients groups are compared using a non-parametric ANOVA Kruskal-Wallis test for trend analysis between groups of rising sepsis severity: ***P < 0.001

Reduced plasma amounts of both S1P carrier proteins, HDL and SA, were characterized as indicators for sepsis severity [27, 28]. In the present study, we also observed a strong correlation between the SOFA score and HDL levels, but not SA levels. Surprisingly, HDL, HDL-S1P, and SA-S1P levels revealed an inverse correlation with the SOFA score, while SA levels did not (Fig. 3). All patients showed similarly reduced SA levels compared to healthy controls regardless of their SOFA score (Fig. 3). Thus, the significant decrease of SA plasma levels was a common observation in all patients, while HDL levels progressively decreased with increasing SOFA score. SA-S1P and HDL-S1P also decreased with increasing SOFA score, but due to the redistribution of S1P from SA to HDL in all patients compared to healthy controls, the contribution of SA-S1P to total plasma S1P was negligible compared to HDL-S1P in all patients in contrast to healthy controls, where SA-S1P had a significant share in total plasma S1P. To further demonstrate the potential of HDL-S1P, SA-S1P, or of the carrier proteins HDL and SA together with various other inflammatory markers to predict either septic shock or sepsis severity with SOFA ≥ 8, a multivariate logistic regression analysis for these parameters was performed. Multivariate logistic regression showed HDL-S1P together with HDL as the strongest and most significant predictors for sepsis severity in this model (*P* < 0.05; Table 3).

**Table 3:** Multivariate logistic regression of HDL-S1P, SA-S1P, S1P carrier proteins (HDL and SA), and various other inflammatory markers as predictors for sepsis severity defined as septic shock or SOFA score ≥ 8

Variable	Regression coefficient	Odds ratio (95% CI)	Significance (*P* value)
HDL-S1P, μg/L	− 0.146	0.86 (0.77–0.97)	< 0.05
SA-S1P, μg/L	+ 0.070	1.07 (0.94–1.23)	ns
HDL, mg/dL	− 0.320	0.73 (0.55–0.97)	< 0.05
SA, g/L	+ 0.166	1.18 (0.85–1.64)	ns
Leucocytes, ×10^9^/L	+ 0.105	1.11 (0.96–1.29)	ns
CRP, mg/L	+ 0.000	1.00 (0.98–1.02)	ns
Interleukin-6, ng/L	+ 0.001	1.00 (1.00–1.00)	ns
Procalcitonin, μg/L	+ 0.254	1.29 (0.87–1.91)	ns

*S1P* sphingosine 1-phosphate, *HDL* high-density lipoprotein, *SA* serum albumin, *ns* not significant

## Discussion

**Fig. 4:**
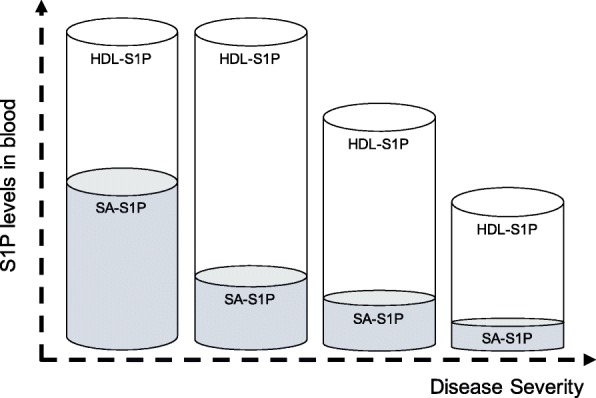
Possible changes of sphingosine 1-phosphate (S1P) sources in blood during sepsis. In healthy controls, S1P is almost equally distributed between serum albumin (SA) and high-density lipoproteins (HDL). In trauma patients with low SOFA score, the total plasma S1P remains constant. However, S1P is shifted from SA to HDL as carrier. In sepsis patients, total plasma S1P levels decrease with higher severity. The significant decrease is explained mainly by the loss of HDL-S1P. The contribution of SA-S1P is insignificant

We found reduced levels of plasma S1P in patients with sepsis, and the levels correlated well with the severity of sepsis-induced organ dysfunction (Fig. 1). Furthermore, we determined the contribution of the responsible S1P carrier proteins SA and HDL to the drop of S1P in sepsis. In the control group, relative values of S1P bound to HDL (45.5%) and SA (35.1%) fit well with reported values for human plasma with 54.1% bound to HDL and 35.6% found in the lipoprotein-depleted plasma fraction [29] In summary, our study unraveled two principal findings regarding alteration of S1P carriers in post-surgery patients, sepsis patients, and patients with septic shock: The first intriguing observation of our study was the redistribution of S1P from SA to HDL in all patient groups compared to healthy controls. This percental shift of S1P carrier towards HDL even in surgical trauma patients with low SOFA score suggests that the drop of SA-S1P can already be detected in disease conditions like post-surgery trauma far away from septic shock. Nevertheless, this redistribution could be an important adaptive response to stabilize plasma S1P at high levels. On the other hand, low amounts of SA-S1P may also be maladaptive and contribute to the pathology of trauma patients, which persists in patients with sepsis and septic shock. This is the first description of this redistribution process, and we currently have no information about its cause, mechanism, and function. Second, the absolute values of both carrier proteins, SA and HDL, behave completely different depending on the severity of disease: While SA-S1P dropped significantly in post-surgery patients and sepsis patients characterized by a low SOFA score, total plasma S1P levels were maintained via increased HDL-S1P levels in these patients. In contrast, the final transition to decreased total plasma S1P in septic shock patients was predominantly caused by a sudden drop of HDL-S1P in this most severe phase of the disease. Figure 4 illustrates these shifts of S1P fractions in patients with rising sepsis severity and organ failure and may serve as a model to further elucidate therapeutic supplementation strategies for S1P in sepsis. SA-S1P was already reduced in conditions like post-surgery trauma that are not necessarily associated with a severe outcome. However, HDL-S1P contributed most to the severe drop of total plasma S1P found in septic shock patients. This is in concordance with reports suggesting that low HDL-S1P contributes to increased sepsis severity [21, 23]. In support of a significant contribution of HDL-S1P to the beneficial effect of HDL, stable fusion proteins of S1P-binding ApoM and the fragment crystallizable region (Fc) of immunoglobulins were successfully used in mouse models of different vascular diseases [30].

Our findings are in line with several experimental and clinical studies reporting a drastic loss of S1P in experimental sepsis models in animals and also in patients with sepsis and septic shock [31]. A decreased amount of S1P in patients had the same predictive value for septic shock as the SOFA score [9]. But quantification of S1P in plasma and serum is currently not a routine method in clinical chemistry. Difficulties regarding extraction, carry-over, and sensitivity issues still need to be solved in order to measure S1P on a routine basis. The main carriers of S1P in plasma however can be easily determined in clinical diagnostics, and our data demonstrate that HDL-S1P levels were reduced similarly to HDL. HDL itself was shown to be a predictive marker for sepsis severity [32]. HDL cut-off values below 20 mg/dL were an independent and sensitive risk-factor for predicting 30-day mortality [33].

Although the share of SA-S1P in total plasma S1P was generally very low in all patient groups compared to healthy controls, it still decreased with increasing SOFA score despite unchanged total SA amounts. The reason for this diminished S1P association with SA is currently not known. Oxidative damage of SA during sepsis could be one reason for a lower binding capacity of SA [34]. Other reasons could also be elevated filtration and removal of SA-S1P from circulation by kidneys, increased diffusion into tissues, and enhanced cellular degradation. Importantly, the mechanisms leading to this effect are not specific for sepsis or septic shock but might begin early and in different conditions of physical stress such during the acute-phase reaction with systemic inflammation after surgery.

While HDL and HDL-S1P showed proportional plasma alterations in patients, loss of SA-S1P was independent from SA levels in patients (Table 2; Fig. 3). This result may be explained by the fact that in contrast to HDL, where S1P is physically bound to ApoM [21], binding of S1P to SA is loose and not efficient [35]. One possible explanation for enhanced, disproportionate loss of S1P from SA could be that SA only increases the solubility of S1P in plasma rather than binding it directly.

Taken together, the observed shift of S1P from SA to HDL occurs early in systemic inflammation. The loss of S1P in sepsis however depends on HDL as carrier protein. These observations are important to evaluate future strategies for the reconstitution of vascular protective S1P in sepsis. One possibility is substitution of human albumin (HA) to septic patients, which is currently intensively debated. Clinical data indicate that HA supplementation has no beneficial effect in sepsis patients, but may have an advantage for patients suffering from septic shock [36]. Our data demonstrate that albumin-bound S1P is particularly low in blood of patients with multiorgan failure and shock. But due to the limited contribution of SA-S1P to total plasma S1P in patients with septic shock, HA supplementation should only have minor effects on total plasma S1P levels in blood, which could be the reason for the limited beneficial effect of HA supplementation observed in patients with septic shock [36]. In contrast, our data suggest that strategies to elevate HDL and HDL-S1P might be superior in stabilizing S1P levels in blood with the goal to profit from the protective effects of S1P when sepsis is most severe [21, 23]. Of note, our data provide evidence that increased HDL significantly correlates with increased HDL-S1P, which is not the case with SA and SA-S1P. Therapeutic strategies to increase HDL may therefore also increase HDL-S1P, which might not be the case for HA and albumin-bound S1P. Defining the reasons for the drop of total S1P levels in plasma of septic patients will be important to understand the underlying regulatory mechanisms and to find better ways to maintain vascular protective effects of S1P in circulation.

Our study has several limitations. It was carried out at a single center and involved relatively small numbers of patients admitted to the ICU. We cannot exclude that our results are biased by sample size or treatment strategies, although we have excluded patients receiving HA. We measured HDL-S1P and SA-S1P and correlated the measured values with clinical and laboratory parameters. But our observations cannot explain cause-consequences at the end. Nevertheless, we believe that our observations warrant follow-up studies with larger patient groups to confirm the power of HDL and HDL-S1P to indicate sepsis severity as well as studies to investigate whether patients with sepsis might benefit from therapeutically elevating HDL levels.

## Conclusion

S1P is mainly associated with HDL and SA in plasma. Surgical trauma patients with a low SOFA score revealed a major shift of S1P from SA to HDL, which resulted in a minor contribution of SA-S1P to total plasma S1P in all patients in contrast to healthy controls (Fig. 4). The observed shift towards increased HDL-S1P also stabilized total plasma S1P levels in surgical trauma patients and sepsis patients at high levels. The massive loss of HDL in septic shock patients with high SOFA score was mainly responsible for the significant decrease in total plasma S1P. Thus, strategies to elevate HDL rather than SA may be an efficient way to stabilize plasma S1P levels in patients with septic shock.

## Abbreviations

ANOVA: Analysis of variance
BP: Blood pressure
CRP: C-reactive protein
EC: Endothelial cells
HDL: High-density lipoproteins
ICU: Intensive care unit
IL-6: Interleukin-6
IQR: Interquartile range
LC-MS/MS: Liquid chromatography coupled to triple-quadrupole mass spectrometry
LDL: Low-density lipoproteins
MRM: Multiple reaction monitoring
PCT: Procalcitonin
RBC: Red blood cells
rcf: Relative centrifugal force
rho: Spearman’s rho
RT: Room temperature
S1P: Sphingosine 1-phosphate
SA: Serum albumin
SOFA: Sequential Organ Failure Assessment
VLDL: Very low-density lipoproteins
